# Coagulation Status Using Clot Wave Analysis in Patients With Prolonged Immobilization

**DOI:** 10.7759/cureus.51483

**Published:** 2024-01-01

**Authors:** Salfarina Iberahim, Rosmaniza Muhamat Yusoff, Noor Haslina Mohd Noor, Rosline Hassan, Noor Nabila Ramli, Rosnah Bahar, Zefarina Zulkafli, Wan Suriana Wan Ab Rahman, Azly Sumanty Ab Ghani

**Affiliations:** 1 Hematology, School of Medical Sciences, Universiti Sains Malaysia, Kota Bharu, MYS; 2 Basic and Medical Sciences Unit, School of Dental Sciences, Universiti Sains Malaysia, Kota Bharu, MYS; 3 Pathology, Hospital Sultanah Nur Zahirah, Kuala Terengganu, MYS

**Keywords:** aptt, pt, prolonged immobilization, cwa, activated partial thromboplastin time (aptt), prothrombin time (pt)

## Abstract

Background

Prolonged immobilization is widely recognized as a risk factor for thromboembolism. In this prospective study, we investigated the changes in clot waveform analysis (CWA) parameters in prolonged immobilized patients following lower limb trauma. CWA is an advanced method for assessing global coagulation that involves continuously monitoring changes in light transmittance, absorbance, or light scattering during routine clotting tests. Additionally, we also aim to determine the CWA parameters between day one and after day three of immobilization.

Methods

A total of 30 patients with prolonged immobilization were enrolled in this study. The plasma of these patients was collected on the first day of their admission and subsequently obtained again after day three of immobilization. Prothrombin time (PT)-based CWA and activated partial thromboplastin time (aPTT)-based CWA were performed using the ACL TOP 300 CTS (Werfen: Bedford, USA) coagulation analyzer, which utilizes the optical method for clot detection. Plasma samples for 20 normal controls were recruited from a healthy blood donor. The CWA parameters generated during clot formation were analyzed. For the comparison of CWA parameters between patients with prolonged immobilization and healthy controls, the Mann-Whitney test was used. A paired t-test was used for the comparison of clot wave parameters between day one and after day three of immobilization. This study was approved by the Universiti Sains Malaysia Research Ethics Committee.

Result

The mean values of PT and aPTT in healthy controls were 11.66 seconds and 33.98 seconds, respectively. There was no statistically significant difference between the patients and the healthy controls in the median values of aPTT (P=0.935). However, patients with prolonged immobilization exhibited significantly higher median PT CWA parameter values than controls (P=0.007). These parameters included the delta change (P<0.001), peak time velocity (P=0.008), and height velocity (P<0.001). On the other hand, the delta change (P<0.001) and height velocity (P<0.001) of the aPTT CWA parameters were significantly higher in patients with prolonged immobilization than in controls. In patients with prolonged immobilization, there was no significant difference in PT CWA parameters between day one and after day three of immobilization, while for aPTT CWA, all parameters were higher on day three, except for the endpoint time.

Conclusion

Patients with prolonged immobilization exhibit increased PT and aPTT CWA parameters compared to normal controls. CWA parameters could aid in identifying patients at risk of developing thrombosis through changes in the clot waveform. However, further study is needed to fully utilize additional information from routine coagulation testing.

## Introduction

Venous thromboembolism (VTE) is a significant health problem, and it comprises both deep vein thrombosis (DVT) and pulmonary embolism (PE). It contributes to one of the most significant causes of mortality and morbidity in hospitalized patients. It is costly to the healthcare system in terms of management, including treatment following VTE. After myocardial infarction and stroke, it is the third most common cardiovascular disease in the Western parts of the world [1].

The overall VTE rates are 100 per 100,000 populations per year, and 70% are hospital-acquired [2]. Approximately two-thirds of VTE episodes manifest as DVT, while the remaining one-third is characterized by PE with or without concurrent DVT. The rates of VTE increase sharply after the age of 45 years and exhibit a slightly greater prevalence in males compared to females [3]. Immobility is a recognized risk factor for VTE, not only in surgical patients but also as a potential risk factor in medical patients. The risk of VTE increases significantly two times higher in immobilized patients than in those with normal mobility [4]. According to Malaysian clinical practice guidelines on preventing and treating venous thromboembolism issued in 2013, immobilization is when a patient is bedridden for more than three days [5,6].

The diagnosis of VTE is confirmed by the radiological examination. DVT diagnosis is established from compressive ultrasonography, while PE is confirmed from the computerized tomography pulmonary angiogram (CTPA). Clinical prediction models are available to help identify patients at risk for VTE, and the most widely used is the Wells' score. DVT is likely if the Wells' score is ≥ 2, and PE is likely when the score is >4.

VTE results from the formation of blood clots in the vein, mainly composed of fibrin and platelet plugs. Current practice to assess the coagulation system is performing the screening tests for coagulation: the prothrombin time (PT) and activated partial thromboplastin time (aPTT). The presence of a hypercoagulable state can be identified by the activation of the coagulation system, leading to a shortened PT and aPTT, and an increase in the levels of D-dimers, fibrinogen, and platelet count [7].

Clot waveform analysis (CWA) provides additional information from routine PT and aPTT testing besides clotting time, and it can be applied in various clinical settings. Many studies focused on the application of CWA on disseminated intravascular coagulation (DIC), congenital bleeding disorders, and sepsis [8,9]. However, there are limited studies examining CWA for PT and aPTT in patients undergoing prolonged immobilization. The biphasic pattern of the aPTT waveform was observed in lupus anticoagulant (LA) positive patients, hemophilia cases with and without inhibitors, and patients with DIC [8]. Suzuki et al. reported that the height of the first derivative and second derivative peaks is useful for diagnosing DIC and predicting the likelihood of hemorrhaging or patient outcomes in individuals afflicted with DIC [10]. Patients with acute VTE exhibited increased aPTT in thrombotic disorders as determined by CWA parameters compared to controls [11]. Another study found CWA values were higher in patients with high Padua Prediction Scores (PPS) [12].

This additional information can be obtained from the routine tests of PT and aPTT without extra cost. However, the PT and aPTT clot waveform parameters are not well utilized to explore other potential uses of CWA parameters. Therefore, in this study, we will evaluate the CWA parameters of PT and aPTT in patients with prolonged immobilization by comparing the CWA parameters between patients and normal controls and identifying the difference in clot waveform parameters at day one and after day three of immobilization.

## Materials and methods

Study design and patients

This prospective cohort study involves patients with prolonged immobilization due to lower limb injuries in the orthopedic ward of Hospital Universiti Sains Malaysia (USM) from November 2020 until October 2021. A total of 30 patients aged from 18 to 48 years old were included. The clinical and laboratory data consisting of age, gender, body mass index (BMI), and smoking status were traced from the patients' folder. The inclusion criteria were adult patients aged between 18-60 years with prolonged immobilization in Hospital Universiti Sains Malaysia during the study period, patients confined to bed for more than three days, and patients with a baseline coagulation profile of PT and aPTT during admission. The exclusion criteria were: patients on anticoagulant or antiplatelet therapy, patients with cancer, recent VTE, thrombophilia, and lupus anticoagulant, DIC, liver disease, coagulopathy, sepsis, and women on hormonal treatment or pregnant. The study also included a control group of 20 healthy individuals free from chronic disease. Ethical approval was given by the Human Research Ethics Committee of Universiti Sains Malaysia (JEPeM code: USM/JEPeM/20010032). 

Blood collection and preparations

Patients with baseline coagulation profiles taken on admission were followed up until repeat samples were taken after day three of immobilization (days four and five). Based on Wells' criteria, immobilization is defined as when a patient is bedridden for more than three days [5]. The blood samples for PT and aPTT tests were collected in a trisodium citrate bottle (2.7 ml of blood) and sent immediately to the Hematology Laboratory Hospital USM. Samples were centrifuged for 15 minutes at 2500 g at room temperature to obtain the platelet-poor plasma (PPP). The PPP was then separated from the whole blood.

Detection of clot waveform for PT and aPTT testing

This study used a conventional coagulometer that utilizes the optical method. The clot waveform was obtained concurrently from the ACL TOP 300 CTS (Werfen: Bedford, USA) coagulation analyzer during routine PT and aPTT testing. This waveform is generated by continuously monitoring light absorption during the clot formation process. The CWA was automatically constructed by plotting changes in light absorbance against time. The parameters that have been studied for the PT are baseline length, time of endpoint, delta change, and first derivative peak (represented by peak time velocity and height velocity) of the clot formation. For aPTT, the baseline length, endpoint time, delta change, first derivative peak (represented by peak time velocity and height velocity), and second derivative (represented by peak time acceleration) were studied. All the parameters were manually recorded and analyzed.

**Figure 1:**
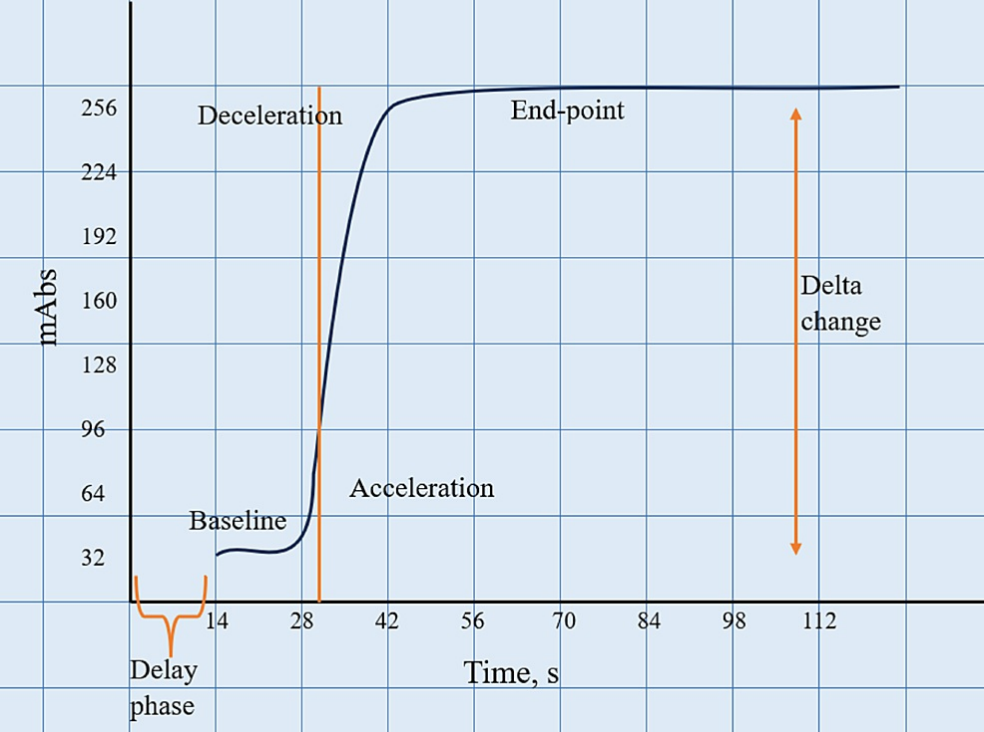
Normal clot signature curve for prothrombin time (PT) and activated partial thromboplastin time (aPTT)

The ACL series coagulation analyzer detects light absorbance during clot formation. The CWA parameters produced by the ACL series consist of the delay phase, baseline phase, acceleration, deceleration, endpoint, and delta change, as shown in Figure 1. The delay phase indicates the mixing process between the sample and the reagent, and the data acquired during this phase is not used for the analysis. The baseline begins after the sample and reagents are mixed. Fibrin formation is represented by the acceleration phase, and the optical change during this phase is rapid, resulting in a steep slope rise. As fibrin formation progresses, the sample becomes more turbid, reducing the amount of light falling on the photosensitive detector. The deceleration phase indicates a reduction in the rate of clot formation. During this time, the fibrinogen has been converted to fibrin, causing the optical change and the slope to plateau. Data acquisition stops at the endpoint. Delta change represents the change in light absorbance between the baseline and endpoints [13].

**Figure 2:**
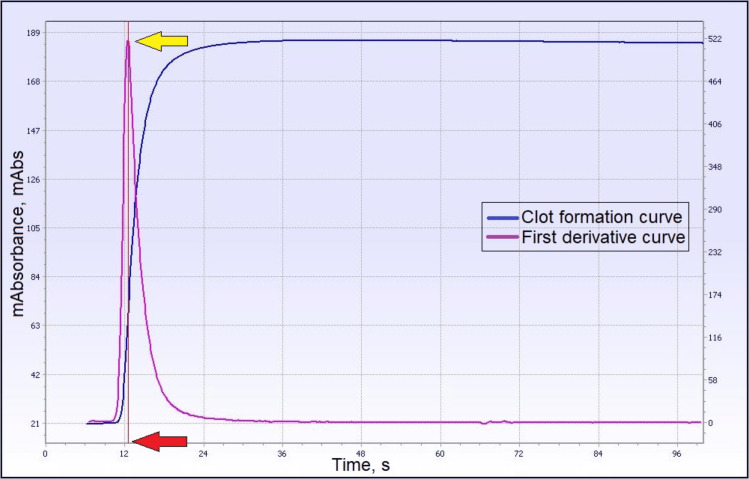
Example of a normal clot waveform for prothrombin time (PT) and its derivative

**Figure 3:**
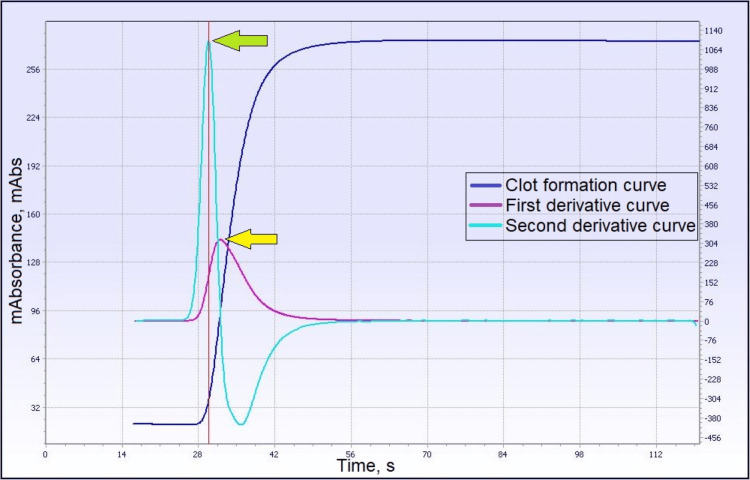
Example of a normal clot waveform for activated partial thromboplastin time (aPTT) and its derivatives

Normal PT clot signature curve has a first derivative curve (purple line in Figure 2) that represents the velocity of the clotting process, which is automatically derived from the software. Peak time velocity (red arrow in Figure 2) is the time at which the maximum velocity of the clotting process is achieved, and height velocity (yellow arrow) represents the amount of light absorbance during the clot formation. The aPTT clot signature curve (Figure 3) provides additional data apart from clotting time, which includes the first derivative (yellow arrow) and second derivative (green arrow) that represent the maximum velocity and maximum acceleration of the clotting process, respectively. Other coagulation analyzers, such as the MDA and Sysmex CS series, detect light transmittance during clot formation. So far, there has been no study of the transmittance curve in the PT produced by MDA and the Sysmex CS series. Most studies focused on the aPTT transmittance curve.

**Figure 4:**
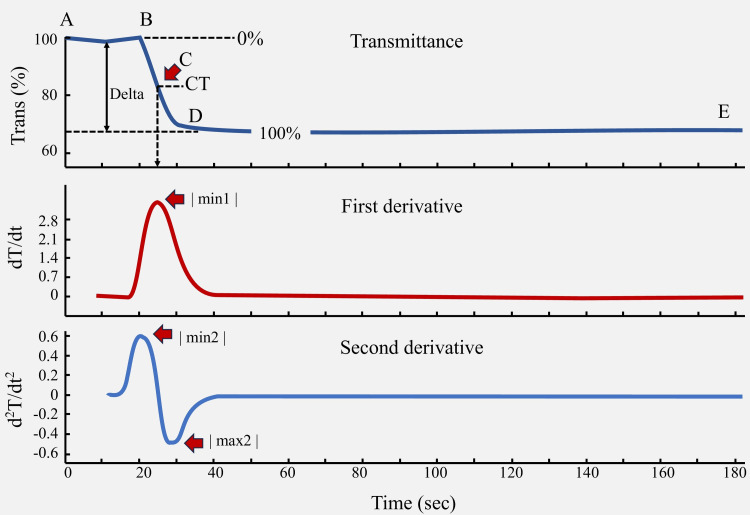
Normal aPTT clot transmittance curve and its derivatives Source: Ichikawa et al., Evaluation of coagulation status using clot waveform analysis in general ward patients with COVID-19, Journal of Thrombosis and Thrombolysis, 2022. Reproduced with permission from Springer Nature Customer Service Centre (SNCSC).

In the aPTT clot transmittance curve, the upper waveform shows the recording of changes in the intensity of transmitted light (T) over time (t) (Figure 4). Point 'A' indicates the beginning of the signal, while point 'B' is the onset of coagulation. Points 'C' and 'D' represent the midpoint and end of the coagulation, respectively. Point E indicates the end of the signal. The data is partitioned into three distinct phases: the pre-coagulation phase (A, B), the coagulation phase (B-D), and the post-coagulation phase (D, E). The red and blue curves were derived from the clot transmittance curve. The middle waveform illustrates the first derivative of transmittance, reflecting the maximum coagulation velocity (min1) that signifies the conversion of fibrinogen to a fibrin clot. Conversely, the lower waveform represents the second derivative of transmittance data that reflects the coagulation acceleration (min2) and maximum coagulation deceleration (max2) [14].

Statistical analysis

Data was recorded and analyzed using the Statistical Package for the Social Sciences (SPSS) Version 25 (IBM Corp.: Armonk, New York). The statistical analysis was performed using the Mann-Whitney test for comparing parameters between patients with prolonged immobilization and normal controls. For the comparison of the clot wave pattern between day one and after day three of immobilization, a paired t-test was used. P values <0.05 were regarded as statistically significant.

## Results

Socio-demographic characteristics of patients

A total of 30 patients with prolonged immobilization aged between 18-48 years old were included in this study. The socio-demographic characteristics of patients are summarized in Table 1. The majority of patients (66.7%) were aged between 18-29 years, with a total of 20 patients falling in this age group. About 23.3% of patients (n=7) were aged between 30-39 years, and the remaining 10% (n=3) were between 40-49 years old. The mean age of the study population was 26.7 years old. Among them, 28 (93.3%) were male and 2 (6.7%) were female. Around 60% of the patients were smokers. Since this data was taken during the movement control order (MCO) period, there were a limited number of patients for this study, and the majority of them were male. All of the patients were of Malay ethnicity and had a normal BMI.

**Table 1:** Socio-demographic characteristics of patients n: frequency; %: percentage; SD: standard deviation; BMI: body mass index.

Variables	Patients with prolonged immobilization (n=30)	
	n (%)	Mean ± SD
Age (Years)		26.7 ± 8.38
18-29	20 (66.7)	
30-39	7 (23.3)	
40-49	3 (10.0)	
Gender		
Male	28 (93.3)	
Female	2 (6.7)	
BMI (kg/m^2^)		21.46 ± 3.96
< 18.5 (Underweight)	8 (26.7)	
18.5-24.9 (Normal)	16 (53.3)	
25.0-29.9 (Overweight)	5 (16.7)	
≥ 30.0 (Obese)	1 (3.3)	
Smoking Status		
Yes	18 (60.0)	
No	12 (40.0)	

Clot waveform analysis for PT and aPTT in healthy controls

The mean PT clotting time for healthy controls was 11.66±0.85 seconds (Table 2). The mean for baseline length was 10.01±0.65 seconds, indicating the time taken to activate the coagulation cascade from adding all reagents up to thrombin production. The mean endpoint for healthy control was 36.39±7.09 seconds. The data acquisition stopped during this time, and all fibrinogens were converted to fibrin. The mean peak time velocity was 11.68±0.83 seconds, which indicates the time taken for the conversion of fibrinogen to fibrin.

**Table 2:** Mean CWA parameter for PT and aPTT of healthy controls SD: standard deviation; CWA: clot waveform analysis; PT: prothrombin time; aPTT: activated partial thromboplastin time.

CWA parameters	Range	Mean ± SD
PT (s)	10.60 – 14.00	11.66 ± 0.85
Baseline length (s)	8.90 – 12.10	10.01 ± 0.65
Endpoint (s)	28.90 – 58.40	36.39 ± 7.09
Delta change (mAbs)	16.00 – 178.50	85.94 ± 35.14
Peak time velocity (s)	11.00 – 14.00	11.68 ± 0.83
Height velocity (mAbs)	49.00 – 199.50	125.96 ± 40.96
APTT (s)	25.80 – 39.00	33.98 ± 3.59
Baseline length (s)	24.20 – 36.60	31.71 ± 3.37
Endpoint (s)	61.50 – 109.80	73.19 ± 13.56
Delta change (mAbs)	20.50 – 247.50	140.50 ± 55.18
Peak time velocity (s)	28.00 – 40.25	36.23 ± 3.46
Height velocity (mAbs)	50.00 – 172.50	117.20 ± 33.82
Peak time acceleration (s)	24.50 – 38.50	33.60 ± 3.66

The mean aPTT clotting time for healthy controls was 33.98±3.59 seconds. The mean baseline length was 31.71±3.37 seconds. The coagulation stopped at 73.19±13.56 seconds. The mean peak time velocity and the mean peak time acceleration were 36.23±3.46 seconds and 33.60±3.66 seconds, respectively.

Clot waveform analysis in patients with prolonged immobilization in comparison with normal controls

Mann-Whitney Test was used in this analysis as the data was not normally distributed. The median PT clotting time for patients was 12.35 (1.25) seconds. The delta change, peak time velocity, and height velocity were significantly higher in CWA for PT in patients with prolonged immobilization compared to healthy individuals (P<0.05). Meanwhile, baseline length and endpoint showed no significant difference between patients and healthy controls (P>0.05). 

The median aPTT for patients was 34.90 (6.18) seconds. The delta change and height velocity were significantly higher compared to controls (P<0.05). Meanwhile, no significant difference was found in the baseline length, endpoint, peak time velocity, and peak time acceleration between healthy individuals and patients with prolonged immobilization (P>0.05) (Table 3).

**Table 3:** Differences in CWA parameters for PT and aPTT between patients with prolonged immobilization and healthy controls Mann-Whitney Test, IQR=interquartile range; PT=prothrombin time; APTT=activated partial thromboplastin time, *P<0.05; **P<0.001

CWA Parameters	Median (IQR)		P-value
	Healthy (n=20)	Patients (n=30)	
PT (s)	11.45 (0.78)	12.35 (1.25)	0.007*
Baseline length (s)	9.95 (0.60)	10.35 (1.57)	0.106
Endpoint (s)	34.65 (4.65)	35.40 (6.52)	0.586
Delta change (mAbs)	85.13 (39.44)	212.50 (96.63)	<0.001**
Peak time velocity (s)	11.45 (0.78)	12.35 (1.25)	0.008*
Height velocity (mAbs)	126.00 (67.88)	242.75 (92.69)	<0.001**
APTT (s)	35.15 (3.80)	34.90 (6.18)	0.953
Baseline length (s)	32.80 (3.57)	32.95 (5.73)	0.744
Endpoint (s)	69.10 (9.32)	69.30 (13.17)	0.242
Delta change (mAbs)	147.00 (69.31)	361.50 (179.81)	<0.001**
Peak time velocity (s)	38.50 (3.50)	36.88 (7.43)	0.873
Height velocity (mAbs)	112.88 (47.50)	210.75 (107.00)	<0.001**
Peak time acceleration (s)	35.00 (5.69)	35.20 (6.60)	0.492

Clot waveform analysis on day one and after day three of immobilization

Mean PT and aPTT after day three of immobilization were 12.46±1.34 seconds and 34.03±3.63 seconds, respectively. For PT CWA parameters, only the endpoint showed a significantly higher level on day one compared to prolonged immobilization (P=0.004) (Table 4). Meanwhile, other CWA parameters for PT showed no statistical differences between day one and after day three of immobilization.

**Table 4:** Differences in CWA parameters of prolonged immobilized patients between day one and after day three of immobilization Paired T-test, SD: standard deviation; PT: prothrombin time; APTT: activated partial thromboplastin time, *P<0.05

CWA Parameters	Mean ± SD		t statistics (df)	P-value
	Day 1	Day 4 and above		
PT (s)	12.63 ± 1.46	12.46 ± 1.34	0.870 (29)	0.391
Baseline length (s)	10.67 ± 1.22	10.26 ± 1.81	1.479 (29)	0.150
Endpoint (s)	38.76 ± 6.09	36.07 ± 4.69	3.123 (29)	0.004*
Delta change (mAbs)	187.97 ± 77.68	215.79 ± 82.48	-1.711 (29)	0.098
Peak time velocity (s)	12.58 ± 1.50	12.46 ± 1.34	0.583 (29)	0.564
Height velocity (mAbs)	220.33 ± 78.18	251.12 ± 80.12	-1.759 (29)	0.089
APTT (s)	32.13 ± 3.88	34.03 ± 3.63	-3.055 (29)	0.005*
Baseline length (s)	29.75 ± 3.47	31.91 ± 3.33	-3.457 (29)	0.002*
Endpoint (s)	68.26 ± 9.48	66.92 ± 7.97	1.086 (29)	0.286
Delta change (mAbs)	297.37 ± 112.65	369.16 ± 131.53	-2.779 (29)	0.009*
Peak time velocity (s)	34.18 ± 4.28	36.13 ± 3.98	-2.629 (29)	0.014*
Height velocity (mAbs)	184.40 ± 63.63	218.56 ± 69.07	-2.184 (29)	0.037*
Peak time acceleration(s)	32.35 ± 4.31	34.04 ± 3.67	-2.645 (29)	0.013*

For the CWA parameters of aPTT, significant differences were observed in the aPTT clotting time, baseline length, delta change, peak time velocity, height velocity, and peak time acceleration of the patients between day one and after day three of immobilization (P<0.05). Meanwhile, endpoints showed no significant difference between day one and after day three of immobilization (P=0.286).

## Discussion

This research investigates the potential efficacy of CWA parameters in identifying a hypercoagulable condition in prolonged immobilization patients who are at risk of developing acute VTE. Patients with prolonged immobilization due to lower limb trauma following a motor vehicle accident (MVA) were involved in this study. The clot waveform parameters of PT and aPTT were sequentially examined in these patients on day one and after day three of immobilization. Four parameters related to the PT curve, namely the baseline length, endpoint, delta change, and maximum velocity, were thoroughly examined and analyzed. Additionally, several aPTT CWA parameters, including the baseline length, endpoint, delta change, maximum velocity, and peak time acceleration, were also subject to study and analysis.

There was no significant difference in the median of aPTT between patients with prolonged immobilization and controls, but the PT of the patient is slightly longer than the controls. Trauma can induce a hypercoagulable state that is frequently manifested by pathological thrombosis [15]. Previous research conducted on specific groups of patients has indicated that a shorter aPTT is linked to a higher risk of VTE [16-18]. The underlying rationale behind this correlation could be attributed to the increased activity of coagulation factors in either the intrinsic or common pathways or potentially due to resistance towards activated protein C [15,17]. Injury due to trauma exposes tissue factors to the circulation and initiates clot formation. The activation of platelets enhances the generation of thrombin and the clotting process, resulting in the consumption of coagulation factors. Additionally, the release of tissue plasminogen activator triggers the activation of fibrinolysis [19]. Excessive consumption of platelets and coagulation factors leads to decompensation, characterized by a decrease in the coagulation factors derived from both the extrinsic and intrinsic pathways, resulting in the normalization or prolongation of PT and APTT [17,18] as seen in this study. This study also noticed a significant increase in CWA parameters of PT among prolonged immobilized patients in terms of delta change, peak time velocity, and height velocity of clot formation. Increased velocity of clot formation means that the clot increased in strength and ultimately had greater density compared to clots in healthy controls [14]. There is little literature on the CWA of PT. A study by Matsumoto et al. suggested that the PT CWA parameters might be useful in predicting and monitoring hemorrhagic symptoms [20]. However, the study on PT CWA in thrombotic disorders is still lacking.

Most of the time, the aPTT waveform was used in the study of the CWA. The clot waveform of the aPTT has been examined in cases of hemophilia, sepsis and disseminated intravascular coagulation (DIC). A study done by Ruberto et al. found that clot waveform analysis could give useful information to recognize a hypercoagulable state in patients with a high PPS. The investigation revealed that elevated aPTT CWA parameters are correlated with higher PPS, thereby indicating its potential as a substitute measure for hypercoagulability. They suggested that the first and second derivatives, aPTT, D-dimer, and fibrinogen levels, could be added to PPS for better assessment of the global thromboembolic risk of the patient [12]. Another study of aPTT CWA utilizing the light transmittance method among COVID-19 patients found that CWA profiles showed a significantly higher mean aPTT, min1, min2, max2, and median delta change than healthy controls. These CWA variables showed hypercoagulopathy, which is more pronounced in critically ill patients [14]. For aPTT CWA, we noted that the delta change and height velocity are higher compared to normal controls. The delta change correlates to fibrin concentration and represents, to some extent, fibrin thickness and clot density. The height of the velocity (first derivative curve) is considered to reflect the 'thrombin burst'. The low height of the velocity curve suggests a bleeding risk and the increased heights of the first and second derivatives curves may be a marker of hypercoagulability [9].

In this study, we found the PT endpoint was higher on day one compared to after day three of immobilization. This may indicate that as the immobilization is more prolonged, the time taken for clot completion becomes shorter, increasing the risk of thrombosis [5]. Other PT CWA parameters showed no difference between the days of immobilization. For the aPTT waveform, all the CWA parameters studied were higher in prolonged immobilization patients except for endpoint time. Prolonged immobilization has been associated with a prothrombotic state. Immobilization of the extremities such as reduced physical activity can lead to stasis of blood in the veins, potentially triggering a hypercoagulable state [21]. This, in turn, may be reflected in the elevated values of the CWA parameters observed in the aPTT waveform, which could potentially indicate an increase in clot formation or an enhancement in clot strength [14].

A recent study among COVID-19 patients found that aPTT-based CWA parameters (maximum velocity, maximum acceleration) became markedly elevated from the second day of admission onward. These markedly raised aPTT-based CWA parameters are possibly consistent with hypercoagulability. The same study also showed that aPTT CWA parameters were markedly raised when the ICU stay was prolonged, suggesting a positive association between rising aPTT CWA parameters and the worsening COVID-19 infection [22].

The aPTT-based CWA parameters have been studied in different types of infections. It has been shown to predict poor outcomes in severe infections with DIC [23]. The biphasic waveform identified in aPTT-based CWA has been extensively investigated in the context of DIC arising from different causes, including infection. This waveform is caused by the formation of calcium-dependent precipitates of very low-density lipoprotein and C-reactive protein (CRP) upon plasma recalcification in vitro. This waveform has been proven to serve as an early indicator of DIC and has been found to be correlated with poor clinical outcomes [23,24].

This study has some limitations. Firstly, the analysis of the PT and aPTT CWA parameters was based on the optical method; hence, the lipemic or haemolyze samples could impact the CWA results and interpretation. Secondly, no other parameters were used to investigate a hypercoagulable state, such as thrombin time and fibrinogen level. Thirdly, a small population of patients was studied. Only a small number of prolonged immobilization patients were not on thrombosis prophylaxis. Most of the patients who were at risk for thrombosis were started on thrombosis prophylaxis early. There was also a limitation in evaluating the clot waveform parameters of the PT and aPTT because it was manually recorded in this study. The development of software in the future for recording the parameters will give a more accurate view of data acquisition.

## Conclusions

In conclusion, our patient with prolonged immobilization showed higher CWA parameters for both PT and aPTT when compared to a control group. Furthermore, individuals with prolonged immobilization demonstrated a higher mean of aPTT CWA parameters after day three of immobilization compared to day one. This study may give clinicians a new insight into the practical utility of CWA in clinical practices. CWA parameters can aid in identifying patients at risk of developing thrombosis through the changes in the clot waveform. However, we need more studies on the clot waveform to fully utilize additional information from routine coagulation testing.

## References

[1] Naess IA, Christiansen SC, Romundstad P, Cannegieter SC, Rosendaal FR, Hammerstrøm J (2007). Incidence and mortality of venous thrombosis: a population-based study. J Thromb Haemost.

[2] Anderson FA Jr, Wheeler HB, Goldberg RJ (1991). A population-based perspective of the hospital incidence and case-fatality rates of deep vein thrombosis and pulmonary embolism. The Worcester DVT Study. Arch Intern Med.

[3] Cushman M (2007). Epidemiology and risk factors for venous thrombosis. Semin Hematol.

[4] Pottier P, Hardouin JB, Lejeune S, Jolliet P, Gillet B, Planchon B (2009). Immobilization and the risk of venous thromboembolism. A meta-analysis on epidemiological studies. Thromb Res.

[5] Wells PS, Anderson DR, Bormanis J (1999). Application of a diagnostic clinical model for the management of hospitalized patients with suspected deep-vein thrombosis. Thromb Haemost.

[6] Zakai NA, Wright J, Cushman M (2004). Risk factors for venous thrombosis in medical inpatients: validation of a thrombosis risk score. J Thromb Haemost.

[7] Zakai NA, Ohira T, White R, Folsom AR, Cushman M (2008). Activated partial thromboplastin time and risk of future venous thromboembolism. Am J Med.

[8] Shima M, Thachil J, Nair SC, Srivastava A (2013). Towards standardization of clot waveform analysis and recommendations for its clinical applications. J Thromb Haemost.

[9] Wada H, Matsumoto T, Ohishi K, Shiraki K, Shimaoka M (2020). Update on the clot waveform analysis. Clin Appl Thromb Hemost.

[10] Suzuki K, Wada H, Matsumoto T (2019). Usefulness of the APTT waveform for the diagnosis of DIC and prediction of the outcome or bleeding risk. Thromb J.

[11] Tan CW, Cheen MH, Wong WH (2019). Elevated activated partial thromboplastin time-based clot waveform analysis markers have strong positive association with acute venous thromboembolism. Biochem Med (Zagreb).

[12] Ruberto MF, Marongiu F, Mandas A, Mameli A, Porru M, Cianchetti E, Barcellona D (2018). The venous thromboembolic risk and the clot wave analysis: a useful relationship?. Clin Chem Lab Med.

[13] Gagnon R (2022). Evaluating the ACL elite series clot signature curve. https://www.scribd.com/document/331264575/ACL-CURVE.

[14] Ichikawa J, Okazaki R, Fukuda T, Ono T, Ishikawa M, Komori M (2022). Evaluation of coagulation status using clot waveform analysis in general ward patients with COVID-19. J Thromb Thrombolysis.

[15] Selby R, Geerts W, Ofosu FA, Craven S, Dewar L, Phillips A, Szalai JP (2009). Hypercoagulability after trauma: hemostatic changes and relationship to venous thromboembolism. Thromb Res.

[16] Tripodi A, Chantarangkul V, Martinelli I, Bucciarelli P, Mannucci PM (2004). A shortened activated partial thromboplastin time is associated with the risk of venous thromboembolism. Blood.

[17] Hron G, Eichinger S, Weltermann A, Quehenberger P, Halbmayer WM, Kyrle PA (2006). Prediction of recurrent venous thromboembolism by the activated partial thromboplastin time. J Thromb Haemost.

[18] Aboud MR, Ma DD (2001). Increased incidence of venous thrombosis in patients with shortened activated partial thromboplastin times and low ratios for activated protein C resistance. Clin Lab Haematol.

[19] Martini WZ (2016). Coagulation complications following trauma. Mil Med Res.

[20] Matsumoto T, Nogami K, Shima M (2014). Coagulation function and mechanisms in various clinical phenotypes of patients with acquired factor V inhibitors. J Thromb Haemost.

[21] Kelsey LJ, Fry DM, VanderKolk WE (2000). Thrombosis risk in the trauma patient. Prevention and treatment. Hematol Oncol Clin North Am.

[22] Tan CW, Low JG, Wong WH, Chua YY, Goh SL, Ng HJ (2020). Critically ill COVID-19 infected patients exhibit increased clot waveform analysis parameters consistent with hypercoagulability. Am J Hematol.

[23] Tan CW, Wong WH, Cheen MH (2020). Assessment of aPTT-based clot waveform analysis for the detection of haemostatic changes in different types of infections. Sci Rep.

[24] Toh CH, Samis J, Downey C (2002). Biphasic transmittance waveform in the APTT coagulation assay is due to the formation of a Ca(++)-dependent complex of C-reactive protein with very-low-density lipoprotein and is a novel marker of impending disseminated intravascular coagulation. Blood.

